# Link between the Reactivity of Slag and the Strength Development of Calcium Aluminate Cement

**DOI:** 10.3390/ma17143551

**Published:** 2024-07-18

**Authors:** Josipa Skočibušić Pejić, Alma-Dina Bašić, Martina Grubor, Marijana Serdar

**Affiliations:** Department of Materials, Faculty of Civil Engineering, University of Zagreb, Andrije Kačića Miošića 26, 10000 Zagreb, Croatia; josipa.skocibusic.pejic@grad.unizg.hr (J.S.P.); alma.dina.basic@grad.unizg.hr (A.-D.B.); martina.grubor@grad.unizg.hr (M.G.)

**Keywords:** calcium aluminate cement, granulated blast furnace slag, R^3^ test, phase assemblage, compressive strength

## Abstract

The problem of loss of strength caused by the conversion reaction with calcium aluminate cements (CAC) is well known. It has been shown that the addition of ground granulated blast furnace slag (GGBS) to CAC inhibits the conversion process. Different slags can have a different chemical and mineralogical composition depending on their origin and production process, which can significantly influence their reactivity. This work investigated the extent to which the R^3^ test, developed for Portland cement and based on isothermal calorimetry and/or bound water, was used to predict the reactivity of ground granulated blast furnace slag in a CAC. Mortars and cement pastes with a 30% replacement of slag were tested to evaluate their compressive strength and microstructure. The results show that slags with higher reactivity due to their hydraulic properties lead to a lower compressive strength loss within the first 6 h, a higher strength loss after 24 h due to stratlingite formation and a lower strength loss after 28 days due to pozzolanic reaction and stratlingite formation. The results also confirm that the R^3^ test was used as a rapid method to predict the effects of slag on the compressive strength of CAC.

## 1. Introduction

Cement production is responsible for 6–8% of CO_2_ emissions. Therefore, the cement industry is facing an increasing demand for supplementary cementitious materials (SCMs). The replacement of cement by SCMs is widespread industrial practice [1,2,3,4]. In the context of reducing CO_2_ emissions, the global and local availability of the SCMs should be considered as an important parameter [5]. One of the commonly used SCM in cement industry is ground granulated blast furnace slag (GGBS), a by-product of the steel manufacturing industry. GGBS has been shown to improve several properties of concrete, such as better long-term properties, including increased chemical resistance together with mechanical resistance and a decreased CO_2_ footprint [6,7]. Blast furnace slag is formed by the combination of earthy constituents of an iron ore with limestone flux [7]. Blast furnace slag major chemical constituents are CaO, MgO, SiO_2_, and Al_2_O_3_ [8]. The chemical composition, and therefore reactivity, of GGBS depends on the quality, nature and proportioning of the iron ore, coke and flux, as well as the fuels used in the blast furnace. The reactivity of a GGBS mainly increases with increasing content of CaO, Na_2_O, and Al_2_O_3_ and with decreasing content of SiO_2_, FeO, TiO_2_, MnO, and MnS [9]. Because of its latent hydraulic properties, GGBS begins to hydrate when it comes into contact with water. In spite of that, this reaction decreases with time and only continues in the presence of an alkaline activator. The GGBSs activation potential depends largely on its properties [7,9]. One of the classical methods to increase slag reactivity is to increase the fineness of the slag [10]. Also, it is considered that the slag reactivity is an important parameter for assessing its suitability as a cement-replacing material in concrete. Thereby, numerous studies aimed to relate reactivity to strength directly, or to develop a correlation which could explain or predict the obtained strength in slag-containing concrete [11]. The generally used way for reactivity determination is compressive strength measurement. This method gives accurate results, but its downside is that it is time and material consuming. Additionally, a range of test methods, which are mostly based on portlandite consumption, are proposed to evaluate the pozzolanic reactivity of SCMs [12,13]. Often, these suggested methods do not correlate well with compressive strength. Therefore, a relatively new method, which is rapid, relevant, and reliable (R^3^) ASTM C1897-20 [8] was proposed for testing a wide range of SCMs’, including GGBS, reactivity in an environment that simulates Portland cement ambience [8,14,15]. The basic principle of the R^3^ test methods is to use a simplified model system to measure the response of an SCM separately from the clinker. This is to avoid interference with the clinker hydration reactions that occur in a blended cement system. R^3^ test proposes two methods to assess SCMs reactivity. The first is by measuring the heat release of the exothermal hydration reactions by isothermal calorimetry, and the second one is the thermogravimetric method that was used to determine the chemically bound water content (from 110 °C to 400 °C) [14]. Isothermal calorimetry is a common method for testing cementitious materials. It is used to determine the very early reactions (first hour or hours), the onset of the main reaction, and the main hydration peak. Heat release is related to the development of most of the properties of cement-based materials. For a specific binder, standard mortar strength development is almost linearly related to the heat release [16]. In previous research [14], a linear correlation of R^3^ test with compressive strength for all ages was detected, both for isothermal calorimetry and for bound water measurement. Blotevogel et al., in their work [15], report that differences in 2 days’ strength of blended cement pastes was predicted by the R^3^ method and that the R^3^ test was highly sensitive to differences in the fineness of the slags. Also, Blotevogel et al. [6] showed that R^3^ calorimetry test was used even to evaluate the impact of minor element additions on GGBS reactivity.

GGBS is commonly used as a replacement for a part of Ordinary Portland Cement (OPC) [17]. A combination of GGBS and calcium aluminate cement is also used in the cement industry [18]. The main phase of CAC is monocalcium aluminate (CA) [19]. During cement hydration, four main calcium aluminate cement hydrates are formed: metastable (CAH_10_ and C_2_AH_8_) and stable (C_3_AH_6_ and AH_3_) hydrates [20,21]. Over time, under the influence of temperature and relative humidity, stable hydrates are formed from metastable hydrates. Metastable hydrates are low density hexagonal phases that fill space at the beginning of cement hydration and thus provide high early compressive strength. During the conversion process, cubic phases of stable hydrates, with higher density, are formed [18,19]. By increasing the density, the porosity of cement matrix also increases, which leads to a decrease in compressive strength [22]. Therefore, even though calcium aluminate cement was originally used to enhance resistance of cement to aggressive sulphates and chlorides, nowadays, CAC cement is used for various applications (quick-setting mixtures, part of expansive component for shrinkage-compensating cements, etc.) [23,24], but its use is limited by the significant loss of strength in the hardened product. The reduction in compressive strength caused by the conversion process was mitigated by the use of chemical and/or mineral additives. The hydration products of CAC cement are changed when a part of the cement is replaced with GGBS. The conversion process may be inhibited due to large amount of SiO_2_ (32–42%) in the composition of GGBS [24]. Silica reacts with calcium aluminate cement hydrates to form C_2_ASH_8_ hydrate (stratlingite or gehlenite hydrate) and reduces the formation of C_2_AH_8_ [18,22,24,25]. Previous studies have shown that the addition of GGBS prevents the reduction in compressive strength caused by the conversion process [18,19,22,23,24,26,27]. For instance, Kirca [26] studied the compressive strength development of mortar depending on the percentage of cement replacement by GGBS. The conversion of metastable hydrates to stable hydrates was not hindered with replacement percentage of 20%, but with increasing the replacement percentage to 40%, 60%, and 80%, strength reduction after 28 days is avoided due to the formation of a sufficient amount of stratlingite. It was shown that the formation of stratlingite prevents the conversion process, and there is no visible decrease in compressive strength over time. The beneficial effect of slag on compressive strength development of CAC was also confirmed by Majumdar et al. [27]. It was reported that replacing the cement with a 50% of slag prevents the reduction in strength caused by the conversion process. In addition to the mechanical properties of calcium aluminate cement, GGBS also affects its fresh properties. The initial setting time of CAC cement is similar to that of OPC, but the time between the initial and final setting is much faster compared to OPC [28]. Yang et al. [18] found that increasing the amount of replacement CAC cement by GGBS prolongs the time between initial and final setting.

One of the challenges of the application of GGBS in CAC remains the availability and the stability of properties of GGBS originating from different sources. It is therefore necessary to have methods that can relatively quickly assess the quality of GGBS and predict the impact of cement substitution on CAC compressive strength development. The objective of the present study is to evaluate whether the reactivity method R^3^ was used for the validation and quality control of GGBS and prediction of compressive strength of CAC-based cementitious composites with GGBS substitution. For this purpose, the influence of industrial GGBS of different reactivity on the properties of CAC mortar were analysed. The reactivity of five industrial GGBS was analysed using R^3^ method (isothermal calorimetry and chemically bound water measurement). The influence of CAC replacement with GGBS was analysed on cement paste and mortar level on five mixes with 30% replacement of CAC with different GGBS. The results obtained were compared with those without GGBS and with those obtained on mix in which 30% of CAC was replaced with the inert material—quartz—as reference mixes.

## 2. Materials and Methods

### 2.1. Materials

Calcium aluminate cement (CAC) used in this research was produced by Calucem d.o.o., Pula, Croatia. The CAC containing 54.9% of Al_2_O_3_ content in the cement was used, of which the C_2_AS phase naturally occurred. Within the scope of the experimental program, 5 different types of slags (GGBS), a by-product of the steel industry, were analysed as possible additions to CAC. The chemical composition of the GGBS, tested by XRF, is given in Table 1. Table 1 additionally gives the loss of ignition (LOI) values, the density tested using the Le Chatelier flask method according to ASTM C-188 [29], mean particle size tested by laser diffraction using the device SALD 3101 (Shimadzu, Kyoto, Japan), Blaine fineness of all used slags, as well as the conformity criteria of ground granulated blast furnace slag for use in concrete, mortar, and grout according to EN 15167 [30].

Based on the chemical and physical properties of GGBS and the criteria given by EN 15167, it was seen that S2, S3, and S4 have higher moisture content than prescribed by the standard. The limits given by EN 15167 for glass content (>66%), MgO content (>18%), and loss of ignition (LOI) (≤3%) were met for all GGBS. Higher amounts of Na_2_O_eq_ were observed for S1, while higher amounts of chloride were observed only for slag S2. Moreover, all tested GGBS had typical ranges of selected properties according to Matthes et al. [9]. In addition to slag, standardised quartz sand (QZ) and tap water were used to produce mortars. Standard quartz sand consists mainly of silicon dioxide SiO_2_ with a relative density of 2.55–2.70 g/cm^3^ at 20 °C. Quartz powder was used as an inert material to distinguish the so-called filler effect from the contribution of a reactive slag.

**Figure 1:**
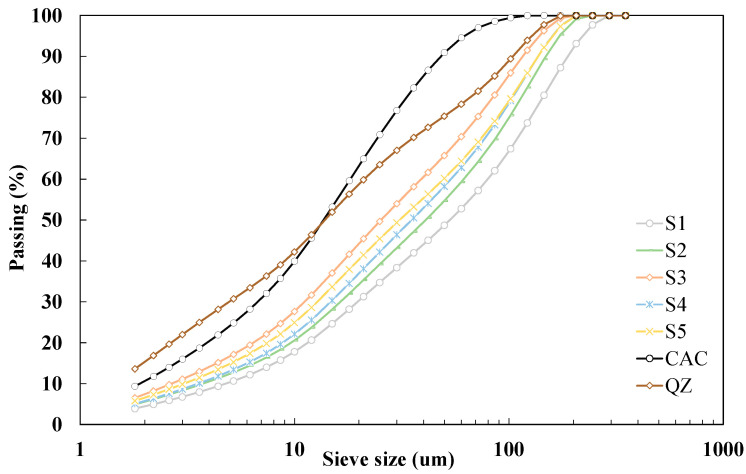
Particle size distribution by laser diffraction on used materials.

Slags were obtained in grains and additionally had varying moisture content due to the physical properties of the material and the relative humidity of the environment in which the slags are stored. Before further use, the slags were dried in an oven at a temperature of 105 °C until the constant weight. After drying, GGBS were milled in laboratory disc mill for 5 min. Figure 1 shows particle size distribution for all studied GGBS. The results show that *d*_50_ of the studied slags is in the range of 26 to 53 μm. All studied slags have coarser finesse (from 1768 to 2668 cm^2^/g) than the values given in the criteria EN 15167 (>3000 cm^2^/g). The density of GGBS ranged from 2.85 to 2.94 g/cm^3^.

### 2.2. Methods

The reactivity of materials was analysed with a test developed for the RILEM TC-267 committee (ASTM C1897-20), called the R^3^ test. Samples of the pastes containing sulphate, alkali additive and SCMs were placed in an isothermal calorimeter at a temperature of 40 °C for 7 days to obtain the total heat release. For each mix, a ratio of Ca(OH)_2_/SCM and CaCO_3_/SCM at 3 and 1/2, respectively, was used, while the alkali solution was 3 M of K prepared with the form of KOH and K_2_SO_4_ as prescribed in ASTM C1897-20 [8]. To avoid temperature fluctuations during measurement, all materials and reagents were weighed, mixed, and kept at 40 °C for 24 h before the experiment. A high shear mixer was used at 1600 ± 50 rpm for 2 min to ensure a homogeneous paste, which was immediately poured into a glass vial and placed in an isothermal calorimeter. Samples of the pastes for bound water measurement were prepared in the same manner as for isothermal calorimetry. The prepared pastes were placed in a plastic container and then cured at 40 ± 2 °C for 7 days in an oven. After 7 days, the pastes were milled in a mortar and dried at 350 °C for 2 h. The bound water was calculated by measuring the change in mass (before and after heating at 350 °C).

Isothermal calorimetry was used to obtain the total heat release for cement pastes and mortars. Analysis was performed by using TAM Air isothermal calorimeter with 8 channels. Seven samples of cement pastes and seven of mortars were prepared: five with a 30% of CAC substitution with different industry GGBS and two referent cement pastes and mortars: a mix with 100% of CAC and a mix with 30% of CAC substitution with inert material—quartz. To prepare the cement paste, the powder was added to distilled water in a plastic vial and mixed by hand until the homogenous paste was achieved. Composition of cement paste was 15 g of CAC and 6 g of water. Samples of the cement pastes were placed in an isothermal calorimeter at a temperature of 20 °C for 24 h. A vial of distilled water was used as a reference. Measurement was carried out according to standard ASTM C1702-17 [31]. Calcium aluminate cement, CEN standard sand [32], and distilled water were used to produce the mortar samples. The composition of the mortars was 13.5 g of CEN standard sand, 5 g of calcium aluminate cement, and 2 g of water. The replacement of calcium aluminate cement with slag was 30% by mass. The mortars were mixed by hand in the plastic vial. Water-to-cement ratio for cement pastes and mortars was 0.4. Mass of cement pastes and mortars used for isothermal calorimetry was 10 ± 0.5 g.

To assess phase assemblage of CAC without and with slag substitution thermogravimetric (TGA) and X-ray diffraction (XRD) analysis were performed. Cement pastes were prepared the same way as for isothermal calorimetry and all samples were cured at 20 °C in desiccator with the relative humidity ~90%. TGA was carried out by TGA 55, TA instruments. The cement paste was ground, and samples of ~50 mg were used. The temperature range was from 30 to 1000 °C, with a heating rate of 10 °C min^−1^, under nitrogen atmosphere. Qualitative XRD analysis was used for the mineralogical identification of crystalline phases in cement pastes. For each sample, the powder of cement paste was packed in a sample holder. XRD was carried out with a Bruker D8 Discover diffractometer using Cu tube with a wavelength of 1.54 Ǻ. The 2θ degrees angle scanned from 5 to 60° with a step size of 0.017°.

Properties in fresh state and mechanical properties of the mortar were investigated for final comparison. The mortar mixes were prepared according to EN 14647 [33]. The composition of the mortar was 1350 g CEN standard sand, 500 g calcium aluminate cement, and 200 g of tap water. The ratio of water-to-cement was 0.4. The replacement of calcium aluminate cement with slag was 30% of the mass. Before casting, the laboratory and all components were conditioned to 20 °C. Temperature, setting time according to EN 480-2:2007 [34], and consistency by flow table according to EN 1015-3:2000 [35] were tested on fresh mortar mixes. After casting, the specimens were covered and stored in metal moulds 40 × 40 × 160 mm in humidity chamber at 20 °C with a relative humidity of 95% for 6 h. After 6 h, the specimens were demoulded and tested for 6 h compressive strength according to EN 196-1:2016 [32]. The specimens for 24 h, 7 d, and 28 d compressive strength testing were stored in tap water at 20 °C in the humidity chamber until testing. To accelerate the conversion samples, the additional set of samples with one chosen slag was cured in water at 38 °C according to EN 14647. The samples were stored in the humidity chamber for the first 24 h. After 24 h, samples were demoulded and placed in water at 38 °C until testing of mechanical properties after 6 and 24 h and 7 and 28 days.

Statistical analysis of the results was performed using the XRealStats (Release 9.1.1) add-in for Microsoft Excel (Microsoft Corporation, Redmond, WA, USA) to quantify the correlation between the compressive strength, reactivity indices, and chemical properties of GGBS with the properties of the CAC system with GGBS. The normality of the data was checked using the Shapiro–Wilk test [28,36]. Based on the results of the Shapiro–Wilk test, i.e., verification that the data are normally distributed, further analyses were performed using Pearson’s correlation coefficients [37].

## 3. Results

### 3.1. Chemical Reactivity of GGBS

**Figure 2:**
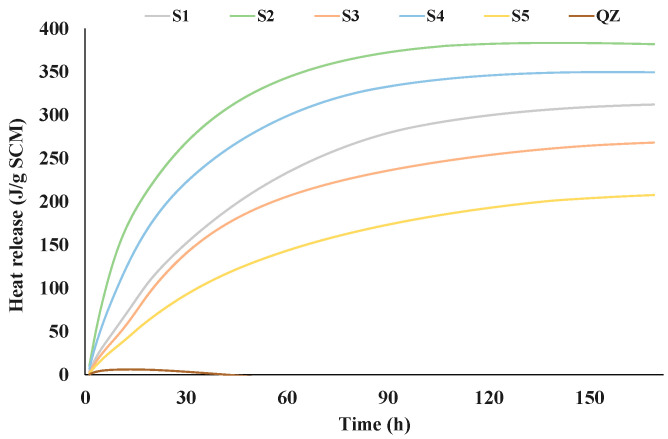
Heat of hydration obtained by R^3^ test using calorimetry during seven days.

**Figure 3:**
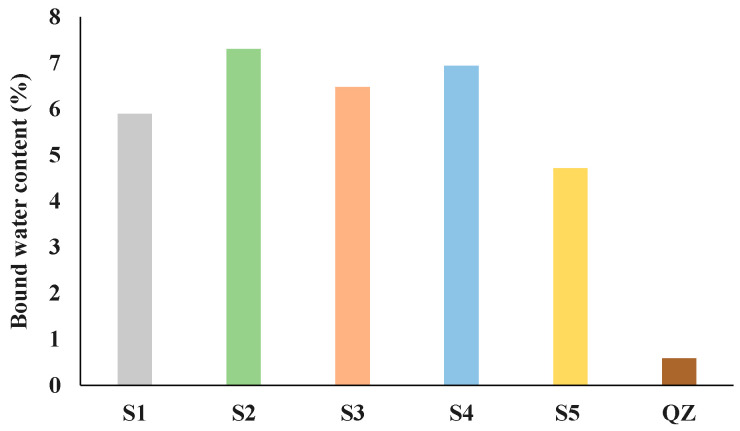
Bound water content for GGBSs obtained by the R^3^ test for reactivity.

Figure 2 and Figure 3 show the results of the R^3^ reactivity tests by isothermal calorimetry and bound water, respectively. The test results of the five different GGBS are shown in comparison with the sample containing inert quartz (QZ).

From the presented results all analysed slags showed higher reactivity than the reference quartz (Figure 2 and Figure 3). The heat release after 7 days for the tested slags ranged from 268 J/g (S5) to 382 J/g (S2). Among all tested slags, the S2 slag showed the best results in the initial reaction. Also, S2 released the highest amount of heat over the 7 days. After S2, S4 then S1 and S3 had the highest heat release. The slag with the lowest heat release was S5. Figure 3 shows the results of the chemically bound water test. The results of chemically bound water show nearly the same trend in the reactivity as the results of the isothermal calorimetry test. The chemically bound water for the analysed slags ranged from 4.71% (for S5) to 7.30% (for S2). According to the results of bound water content the reactivity of the slags decreased down the series as follows: S2, S4, S3, S1 and S5.

### 3.2. Effect of Slags on CAC Paste and Mortar Properties

**Figure 4:**
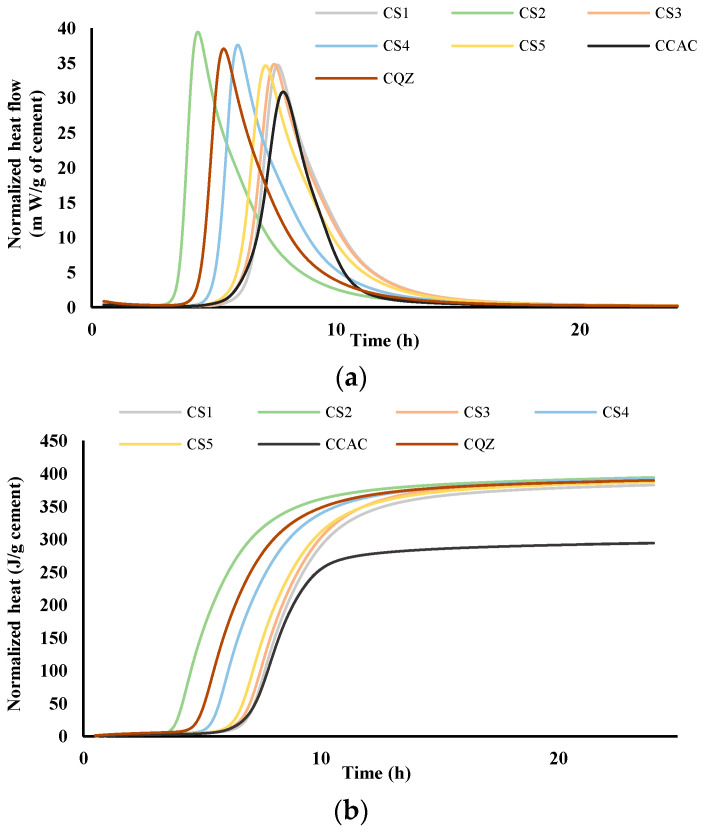
Heat flow, m W/g cement (**a**) and heat release, J/g cement (**b**) in dependence of time for cement pastes; results are normalised per gram of cement.

**Figure 5:**
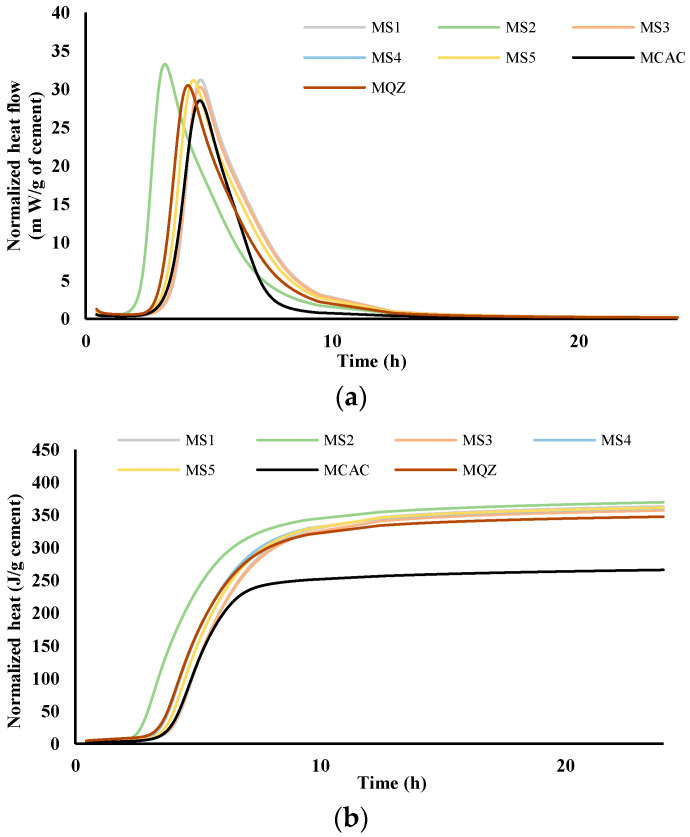
Heat flow, m W/g cement (**a**) and heat release, J/g cement (**b**) in dependence of time for mortars; results are normalised per gram of cement.

The comparison of heat flow for cement pastes (labelled C) and mortars (labelled M) without and with the addition of different slags was performed via isothermal calorimetry at 20 °C. Figure 4 and Figure 5 show heat flow (a) and total heat release (b) normalised per gram of cement during the first 24 h for cement pastes and mortars, respectively.

From the results for total heat release (Figure 4b and Figure 5b), it was seen that in the CAC/slag system, the most reactive system is CS2, i.e., MS2, which compared to CCAC, i.e., MCAC shows an increase in heat release of 34% in cement paste, i.e., 38.9% in the mortar. Comparing the same data with the cement paste and mortar with quartz replacement (CQZ and MQZ), it can also be concluded that slag S2 increases the heat release of the cement not only by the effect of the inert filler, but also chemically. Except for slags S1 and S5 in cement pastes, the slags show that they increase the heat release in the same way as slag S2, i.e., by the action of the inert filler and chemically both in the cement pastes and mortars. In this case, slag S3 increases the heat release by 33.4%, slag S4 by 33%, and S5 by 31.7%. In cement pastes, slag S1 and S5 increase the heat of hydration by 30, i.e., 31.7%, which is slightly less than the quartz-containing system (increases the heat of hydration by 32% compared to pure CAC). Here, however, it must be highlighted that the quartz used was ground finer than any of the used slags, which could lead to a more significant filler effect of quartz, compared to any of the tested slags.

From the graphs showing the heat flow as a function of time (Figure 4a and Figure 5a), it is evident that in the terms of the hydration reaction rate, the slag S2 in the cement paste and in mortar contributes the most. This further confirms the highest reactivity of S2 slag in the vicinity of calcium aluminate cement, which agreed with the results from the R^3^ test (Figure 2 and Figure 3). In cement pastes, the contribution of slags to the heat flow of the main reaction decreases as follows: S2, S3, S4, S5, and S1. In mortars, the contribution of the slag to the heat flow of the main reaction is not the same. After slag S2, the slags contribution decreases as follows: S4, S5, S3, and S1.

**Figure 6:**
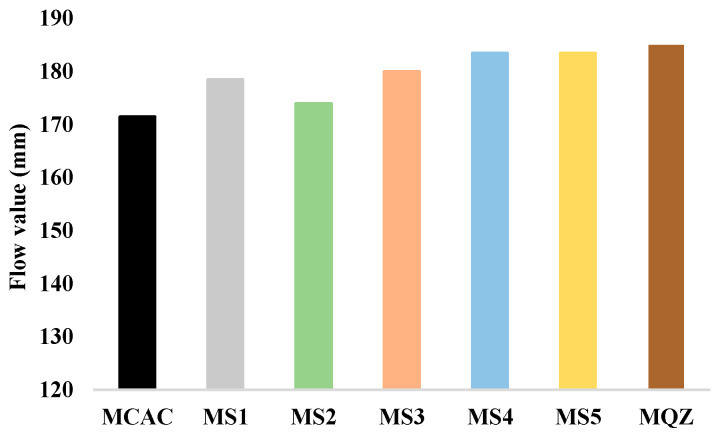
Flow values of fresh cement mortar samples.

The results of the workability of fresh mortar are shown in Figure 6. From the results of the workability of fresh mortar, the substitution of 30% CAC with slag resulted in a slight increase in the flow value for all slags used. An increase in the flow value was also obtained with the addition of quartz. MS2 had the lowest value among all mortars with slag, which could potentially be attributed to the higher water absorption of slag S2, influencing the effective water-to-cement ratio. However, S2 slag has coarser particles compared to other slags, which should also be taken into account when attributing the effect to water absorption.

**Figure 7:**
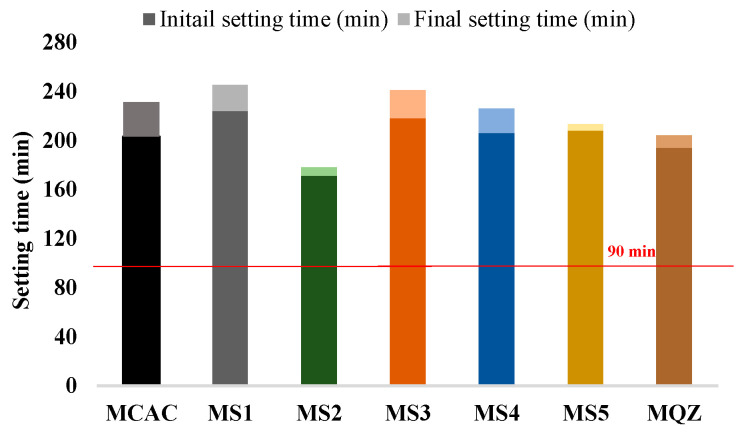
Initial and final setting time of fresh mortar.

According to EN 14647, the setting time for CAC should not be less than 90 min (indicated in Figure 7). From the results of the initial and final setting time, all the mixes met the requirements of the standard. Moreover, all the mixes with 30% slag replacement had a prolonged setting time except the mix with S2 slag.

**Figure 8:**
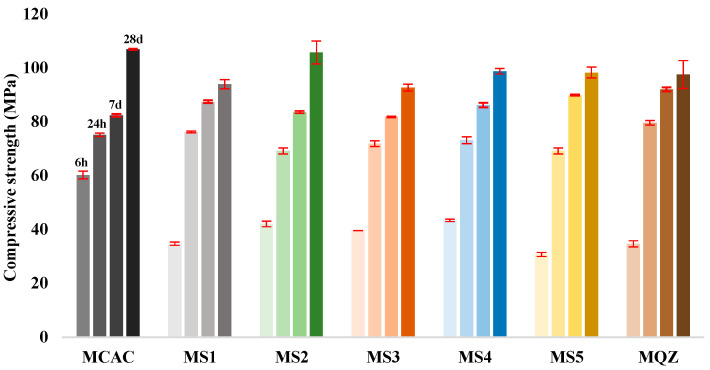
Compressive strength of mortars of the following ages: 6 h, 24 h, 7 days, and 28 days.

The compressive strength measurements were carried out on the mortar specimens after 6 h, 24 h, 7 days, and 28 days (Figure 8). The results showed that at age of 6 h, the studied mixes developed between 31.3% (for MS5) and 56.3% (for MCAC) of their 28-day compressive strength, while after 24 h, the compressive strength development ranged from 65.5% (for MS2) to 81.6% (for MQZ). Moreover, after 7 days the compressive strength development was in the range of 77.0% (for MCAC) to 94.35% (for MQZ) of their 28-day compressive strength.

Compared to the reference mix CAC, the replacement of CAC with slag reduced the compressive strength by 30–50% after 6 h. After 24 h, slag started to contribute to the development of compressive strength and a negligible difference in compressive strength (less than 8%) was observed compared to the reference mix CAC. Moreover, after 7 days, mixes with 30% CAC replaced by slag had slightly higher compressive strength than mixes with 100% CAC (Figure 8).

After 28 days, the compressive strength values of the tested mixes ranged from 92.66 MPa (for MS3) to 106.89 MPa (for MCAC). For these mixes, it was observed that the replacement of CAC with 30% slag decreased the compressive strength by 1–13% after 28 days, while replacement of CAC with 30% of quartz powder decreased the compressive strength by 9%. Mixes MS2, MS4, and MS5 showed higher compressive strength in comparison to mix QZ. It was noticed that 30% replacement of CAC with MS2 slag did not affect (only 1% difference) the compressive strength for later ages (≥24 h) but decreased at early ages (<24 h).

### 3.3. Correlation between Reactivity and Compressive Strength

Pearson’s correlation coefficients were used to preliminary evaluate the significance of the correlation between the R^3^ test for slag, i.e., the heat release and the bound water content, and the compressive strength of CAC with slag addition (Table 2). The main objective of determining the significance of the Pearson’s correlation coefficients for heat release, bound water, and compressive strength was to evaluate whether the R^3^ reactivity method was used for the validation and quality control of slags and the prediction of the compressive strength of CAC cementitious composites with slag substitution. In addition to the Pearson’s correlation coefficients for the R^3^ test results and compressive strength, the same coefficients were calculated for different reactivity indices of GGBSs (from the literature [9]) and compressive strength.

Table 2 shows a summary of the r-values calculated from Pearson’s correlation for different reactivity indices of slags, including heat release (6 h, 24 h, 3 days, 7 days) and bound water content obtained in this research using R^3^ method and the compressive strength (6 h, 24 h, 7 days, and 28 days) of CAC mortars. Reactivity indices are commonly used to assess GGBS quality [15]. While such indices may be practical for the quality control of GGBS from a single source, they are not satisfactory for general prediction of the strength of different origin GGBS in cement because there is no linear relationship between strength and oxide content in GGBS over a wide range of compositions [9]. Still, to assess quality control in this work, different reactivity indices were calculated for a set of five different GGBS based on the oxide contents of the GGBS, using formulas commonly found in the literature [9,15] and shown in Appendix A Table A1.

It was observed that all slag samples had satisfactory criteria for reactivity according to the criteria given by the European Standards for cement and blast-furnace slag I and II, German Standard for “Hochofenzement”, and German Standard for special cements. Also, all slag samples, except S3, had satisfactory criteria for reactivity according to the German Standard for “Hochofenzement”. Criteria given for F-value according to Keil was satisfactory for slags S2, S4, and S5 while only S2 and S4 had satisfactory criterion given in German Standard for “Eisenportlandzement”. According to [9], the more reactivate GGBS had a CaO/SiO_2_ (reactivity index according to Tetmajer) ratio of ≈1.2 and created a far higher strength gain than the GGBS of low reactivity, which had a CaO/SiO_2_ of ≈0.9. In this study, it was found that slag S2 with a CaO/SiO_2_ ratio of 1.2 produced the mortars with the highest compressive strength, i.e., it was the most reactive slag according to the R^3^ test. Slag S4, with a CaO/SiO_2_ ratio of 1.14, was the second most reactive, considering the compressive strength and R^3^ test results (Figure 2 and Figure 3). In addition, the CaO/SiO_2_ ratio was 0.91, 0.87, and 1.09 for S1, S3, and S5 slags, respectively. According to these values, it could be assumed that slag S5 was the third most reactive, but it was shown that slag S5 was the least reactive among all slags. However, the Pearson’s correlation coefficient for the CaO/SiO_2_ ratio and compressive strength of calcium-aluminate mortar with slag substitution after 28 days had an r-value of 0.93 with a *p*-value of 0.01 (Table 2), which meant that it was good for predicting later compressive strength ages of calcium-aluminate cement with the addition of slag. In addition to the CaO/SiO_2_ ratio, the SiO_2_ content also had a high r-value (r-value −0.86, *p*-value 0.03) and it was inversely proportional to the compressive strength of calcium-aluminate cement with slag addition at 28 days. In addition to the SiO_2_ content, the slag density, and the German standard “blastfurnace cement” were also inversely proportional to the compressive strength of calcium-aluminate cement with slag addition after 28 days. Table 2 shows that the German standard for blast furnace cement and for special cements, the reactivity according to De Langavant and according to Wang, the basicity according to Schwiete, the European standards for cement and blast furnace slag, the F-value and the CaO; the CaO/Al_2_O_3_ content in slags offer a relatively good possibility to predict the 28-day compressive strength of calcium-aluminate cement with slag addition (Pearson’s correlation coefficient between 0.60 and 0.93). Overall, although none of the calculated reactivity indices were able to predict early mortar compressive strength, they were effective in accurately predicting 28-day compressive strength.

According to the results presented in Table 2, there was a high correlation between heat release and compressive strength after 6 h, with correlation coefficients (r-values) between 0.75 and 0.84 and an average *p*-value of 0.05. In addition, a strong correlation was found between the bound water content and compressive strength after 6 h, with an r-value of 0.96 and a *p*-value of 0.00. The Pearson’s correlation coefficient between the Al_2_O_3_ ratio and the compressive strength of calcium aluminate mortar with slag substitution after 6 days showed an r-value of −0.92 with a *p*-value of 0.03 (Table 2). This indicated a strong inverse relationship between the Al_2_O_3_ content and the compressive strength of calcium aluminate cement with slag addition after 6 days. Specifically, higher Al_2_O_3_ contents of used slag led to a lower compressive strengths of calcium aluminate cement at early ages. The Pearson’s correlation coefficient between the heat release after 6 h and the compressive strength of calcium-aluminate mortar with slag substitution after 28 days showed an r-value of 0.84 with a *p*-value of 0.04 (Table 2). This indicated a high correlation, suggesting that a higher heat release of slag in R^3^ test in the early stages (6 h) correlated well with a higher compressive strength of calcium-aluminate cement with slag addition after 28 days.

These results indicate that the R^3^ reactivity method is effective for the validation and quality assurance of slags, as well as for predicting the compressive strength (both at 6 h and 28 days) of calcium aluminate cement (CAC) with slag substitution. According to the calculated Pearson correlation coefficients, the reactivity indices are also effective for predicting the 28-day compressive strength of calcium aluminate cement (CAC) with slag substitution.

Additionally, in previous research [6,9,15] it was found that the presence of minor components can have a significant influence on slag reactivity. Especially critical is TiO_2_, which significantly reduces GGBS reactivity and thus strength development at all ages. In this research, it was confirmed that slags with higher TiO_2_ content (Table 1) had lower reactivity (Figure 2 and Figure 3).

### 3.4. Effect of Slag Reactivity on CAC Phase Assemblage

Based on the reactivity results, heat release, workability, and compressive strength of mortars for the further investigation of slag effect on CAC/slag system phase assemblage, slag S2 was chosen as the most reactive and slag S5 was chosen as the least reactive. As a reference, the cement paste without slag substitution (CCAC) was studied. Phase assemblage was studied by thermogravimetric analysis (TGA) and qualitative X-ray diffraction (XRD).

**Figure 9:**
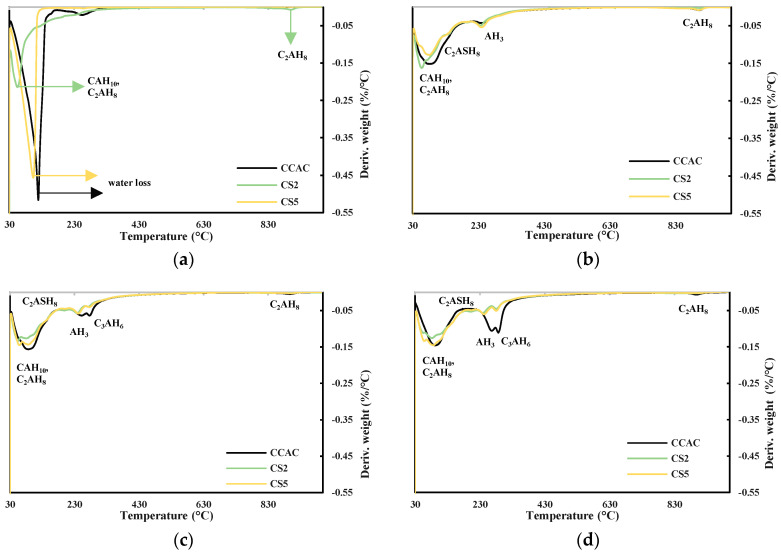
DTG for cement pastes after (**a**) 6 h, (**b**) 24 h, (**c**) 7 days, and (**d**) 28 days.

**Figure 10:**
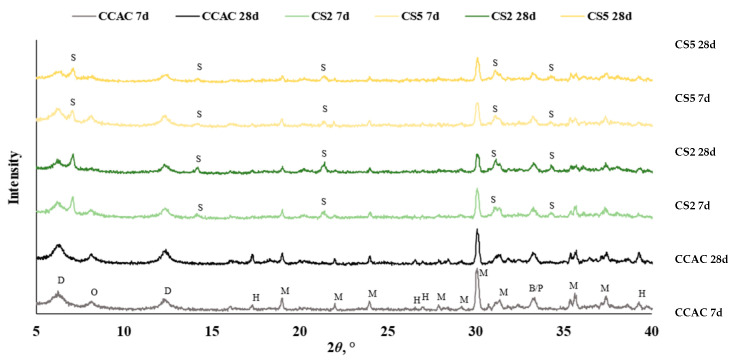
Diffractogram for cement pastes after 7 and 28 days without and with slag substitution CCAC, CS2, and CS5 (B = C_2_S, D = CAH_10_, M = CA, H = C_3_AH_6_, O = C_2_AH_8_, P = CT, S = C_2_ASH_8_).

Obtained results from TGA and XRD are shown in Figure 9a–d and Figure 10, respectively. Figure 9a shows DTG for cement pastes CCAC, CS2, and CS5 after 6 h. For CS2 in the temperature range 30–115 °C, the indication of formed metastable phases CAH_10_ and C_2_AH_8_ formation is visible. Also, for CS2 at 900 °C, there is probably a visible C_2_AH_8_ decomposition. Going backing to this statement, Scheinherrová and Trník [38] performed simultaneous DSC/TG analysis on Secar71 calcium aluminate cement and observed a peak above 800 °C, which they designated to C_2_AH_8_. The sample with lower reactivity slag, CS5, showed a very similar DTG curve to CCAC. After 24 h, for all samples, there was a visible decomposition peak in the temperature range 30–150 °C (Figure 9b), which is assigned to the metastable phases CAH_10_ and C_2_AH_8_. The CS2 sample showed the highest amount of metastable phases. Compared to 6 h for CCAC, CS2 and CS5, the formation of the new peak in the temperature range 213–256 °C designated to the AH_3_ was detected. Additionally, at 24 h, for samples CCAC and CS5 in temperature range 886–923 °C, the decomposition of C_2_AH_8_ was visible. At 24 h, the sample CS2 already depleted C_2_AH_8_ in that temperature range, which was transformed to stratlingite; C_2_ASH_8_ was visible in the temperature range 191–206 °C. At 24 h, sample CS5 showed only a small indication of formed stratlingite while sample CCAC did not form any stratlingite. From 24 h (Figure 9b) to 7 days (Figure 9c), for all samples, a significant increase in the formation of AH_3_ and hydrogarnet with *T*_max_ at 244 °C and 278 °C, respectively, was detected. Compared to the 24 h sample, CS2 showed an increase in the amount of formed stratlingite (*T*_max_ at 198 °C). Additionally, compared to CS2 at 7 days, sample CS5 showed a less intense peak of C_2_ASH_8_. Since slag S2 was more reactive, this behaviour was expected. Regarding CS2 and CS5 at 28 days (Figure 9d), CCAC showed a significant increase in formation of AH_3_ and C_3_AH_6_. At 28 days compared to 7 days, sample CS2 showed an increase in the amount of formed stratlingite, while CS5 showed an increase of AH_3_ and C_3_AH_6_. Therefore, from TGA results up to 28 days, the more reactive slag S2 showed higher quantities of formed stratlingite. The slag with lower reactivity, S5, showed relatively the same behaviour but with a certain delay. This agreed with the predictions of the R^3^ test results. The sample without slag CCAC did not show any formation of stratlingite. Therefore, the influence of slag addition on CAC phase assemblage is evident.

From DTG, the results of samples with slag substitution, CS2 and CS5, overlapping peaks designated to metastable phases were visible, while for CCAC those phases were intertwined, and metastable phases were laying under the same peak. This accentuates the challenges of TGA data analysis for this CAC/slag system as well as the need to use a complementary analytical technique, such as XRD, in order to confirm occurring phases. Hence, for the further investigation of phase assemblage of CAC/slag systems qualitative XRD was performed. In this stage of research, XRD analysis was performed after 7 and 28 days of hydration.

For all samples (CCAC, CS2 and CS5) at 7 days and 28 days (Figure 10), anhydrous phases of CAC were detected: monocalcium aluminate (CA), belite (C_2_S), and possibly perovskite (CT). Additionally, at the diffraction angle 2*ϴ* between 5 and 18°, metastable phases CAH_10_ and C_2_AH_8_ were detected. In all cement pastes, stable hydrogarnet (C_3_AH_6_) was identified with the most intense peak at 2*Ɵ* 39°. The detected formation of C_3_AH_6_ confirmed that a conversion reaction took place, i.e., that hexagonal metastable phases transformed to the stable, more dense cubic phases.

At 7 days, CS2 and CS5 showed the same anhydrous phases (CA, C_2_S and CT) but with lower intensities. Since CCAC had 30% more calcium-aluminate cement in the mix, that was expected. The same trend was seen for metastable phases (CAH_10_ and C_2_AH_8_); after 7 days of hydration compared to CCAC samples, CS2 and CS5 showed lower intensities. Also, CS2 and CS5 showed lower intensities of hydrogarnet phase. With XRD at 7 days, the formation of stratlingite and C_2_ASH_8_ with main peaks at diffraction angle 7, 14, 21, 31, and 34° were confirmed. Comparing CS2 to CS5, it was observed that CS2 had a higher intensity of stratlingite peaks. This indicated good correlation with TGA results (Figure 9) and R^3^ test prediction of slag reactivity (Figure 2 and Figure 3).

From the diffractogram at 28 days of hydration (Figure 10), for CCAC, there was a noticeable increase in the intensity of C_3_AH_6_ peaks. Compared to CCAC at 28 days, CS2 and CS5 had lower intensities of C_3_AH_6_ peaks. Also, for CS2 and CS5, it was apparent that, with prolonged hydration time (from 7 to 28 days), the intensity of stratlingite peaks increased. Still, compared to CS5, the sample CS2 had higher intensities of stratlingite. This trend is the same as in the diffractogram after 7 days and is in good correlation with TGA results.

### 3.5. Effect of Slag on CAC Conversion Process

**Figure 11:**
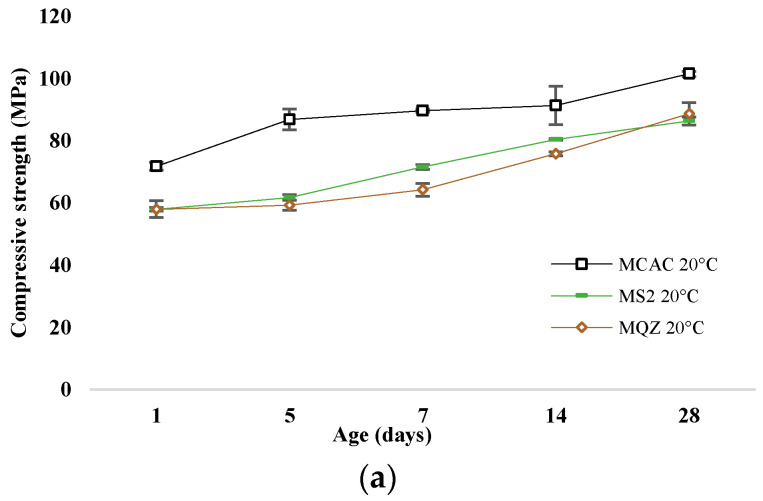

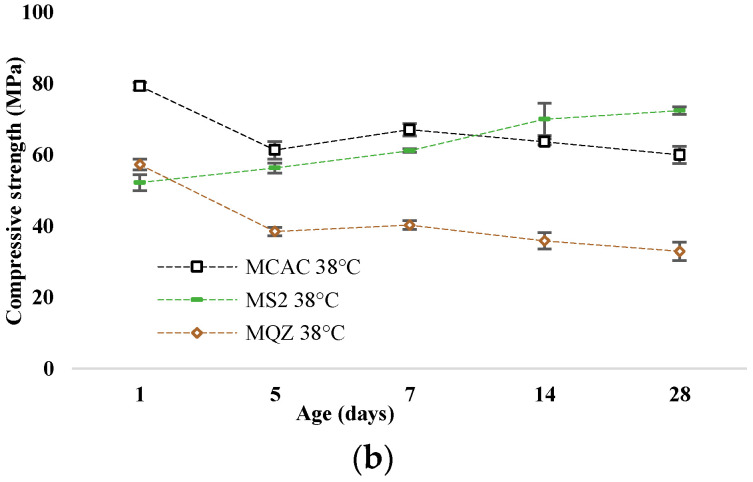
Compressive strength development for samples cured at (**a**) 20 °C and (**b**) at 38 °C.

The most reactive slag was S2; therefore, the mortar with 30% replacement of CAC with slag S2 (mortar MS2) was chosen for a more detailed analysis of the conversion process. The development of compressive strength of mortars without and with slag substitution cured at 20 °C and 38 °C are shown in Figure 11a and Figure 11b, respectively.

After 24 h, the compressive strengths were similar for all mixtures. In the case of the reference mixture, the conversion of metastable to stable hydrates occurred on the 5th day when cured at 38 °C, which is evident from the sharp decrease in strength. This decrease was not evident when cured at 20 °C; on the contrary, the strength constantly increased. The compressive strength of the MQZ mixture continuously increased when cured at 20 °C, while there was a decrease on the 5th day when cured at 38 °C. It was concluded that quartz as an inert material did not interfere with the conversion of calcium aluminate cement hydrates. On the contrary, the MS2 mixture had a constant increase in strength regardless of the curing temperature and no sharp decrease was observed for 28 days when cured at 38 °C.

## 4. Discussion

Slags with higher reactivity yielded mortars with higher 6 h strength and the mortar that had the lowest loss of strength after 28 days compared to MCAC (Figure 8). However, mortars with more reactive slags showed lower compressive strength after 24 h (Figure 11). From DTG results of cement pastes after 6 and 24 h, it was visible that at 6 h, cement paste CS2 showed already formed metastable phases, which contributed to the high compressive strength. Also, CS2 at 6 h showed *T*_max_ at 900 °C of C_2_AH_8_ decomposition while CCAC and CS5 showed only water loss and no evidence of any hydration phases formation. At 24 h of age, that peak with *T*_max_ at 900 °C was visible for CCAC and CS5. Therefore, hydration reactions in those samples were delayed compared to CS2. At 24 h, CS2 already depleted the phase at 900 °C for stratlingite formation (191–206 °C), which did not contribute to the compressive strength as metastable phases. This behaviour agreed with the setting time of mortars (Figure 7), where it was shown that the sample MS2 had the fastest final setting time in comparison to other mortars. Additionally, from the heat flow of mortars (Figure 5a) it was visible that MS2 had the fastest onset of the main reaction and main hydration peak at around 3.27 h. Therefore, a more reactive system like MS2 will react, i.e., form metastable phases, earlier compared to other mortars. Consequently, MS2 will also start to take part in the conversion reaction and transformation of CAH_10_ and C_2_AH_8_ to stratlingite first, which leads to lower compressive strength [39]. On the other hand, MCAC and MS5 will still be in the formation process of metastable phases, which contribute to compressive strength; therefore, they will have higher compressive strength at 24 h. Moreover, Goergens and Goetz-Neunhoeffer [40] stated that CAH_10_ persists for longer period than C_2_AH_8_ before it converts to stable hydrates, which is in agreement with the early decomposition of C_2_AH_8_ for more reactive CS2 and later for CS5. After 28 days, the MS2 again has the highest compressive strength among the mortars with slag replacement and only 1% of compressive strength loss compared to MCAC. Therefore, the behaviour of lower compressive strength for more reactive systems at 24 h was explained by competition between the hydraulic reaction and conversion reaction.

In their research, Blotevogel et al. [15] stated that the chemical composition of liquid slag governs the formation of the glass network. The higher the content of network modifiers such as Ca, Mg, K, and Na, the lower the polymerization degree of the glass network, which results in a less stable slag. Less stable slag will have higher dissolution rate and consequently a faster liberation of ions to the liquid phase, which precipitates to give secondary minerals that may develop material strength [15]. In this case, the secondary forming phase from GGBS is stratlingite. Therefore, higher reactivity results in faster stratlingite formation. In this research, it was confirmed that with an increased amount of TiO_2_ in slags, mortars had lower compressive strengths after 28 days and lower slag reactivity. Also, it was shown that a higher value of CaO/Al_2_O_3_ and a higher amount of CaO had a positive impact on slag reactivity, while the content of Al_2_O_3_ influenced slag reactivity negatively. Additionally, mortars with a higher content of SiO_2_ in GGBS had lower compressive strength of calcium-aluminate cement after 24 h. This is because at 24 h, at the expense of metastable phases, stratlingite started to form and stratlingite did not contribute to the compressive strength.

## 5. Conclusions

The main objective of the present study was to evaluate the applicability of the R^3^ test to the reactivity of GGBS with CAC systems as well as to evaluate the impact of the reactivity of slags on the mechanical properties of CAC mortars with cement substitution with slags. The study researched the GGBS effect on compressive strength on CAC/GGBS mortars, as well as the effect of GGBS on microstructure of cement pastes. The study showed that the slags which were ranked as more reactive, using the R^3^ test method, when used as 30% cement replacement in CAC mortars, exhibited higher 6 h strength and the lowest loss of strength after 28 days compared to the CAC mortar without slag substitution. In addition, it was shown by TGA and XRD that the slag that exhibited higher reactivity in the R^3^ test formed more stratlingite in cement pastes, i.e., inhibited transformation to a greater extent. Therefore, a more reactive slag is more willing to participate in the hydration reaction of calcium aluminate cement.

Compared to all other available reactivity indices, the heat release and bound water measurement according to R^3^ protocol, showed the highest statistical correlation with compressive strength of CAC mortar, both at 6 h and 28 days. From the preliminary results of the Pearson’s correlation that was performed, the strongest linear relationship between compressive strength and bound water was found at age ≤ 6 h with r-values greater than 0.9 (*p*-value 0.00), indicating that measuring the bound water content is a promising method to verify the reactivity of slags in the CAC system at an early age. Finally, isothermal calorimetry protocol from R^3^ test was shown to be the only method that can predict early compressive strengths (≤6 h) and later compressive strength, at 28 days. Thus, using the R^3^ test, first and foremost developed for Portland cement, and the protocol for isothermal calorimetry and the determination of chemically bound water content, the differences in slag reactivity was determined and the impact of cement substitution by slag on mechanical properties of CAC was predicted.

## Figures and Tables

**Table 1:** Chemical composition and physical properties of used slags.

Property	Unit	S1	S2	S3	S4	S5
SiO_2_	%	38.2	35.4	39.8	36.6	35.6
Al_2_O_3_	%	12.1	11.7	12	10.8	13.5
TiO_2_	%	0.6	0.6	1.4	0.5	1.2
MnO	%	1.8	0.2	1.6	0.4	0.3
Fe_2_O_3_	%	0.3	0.5	0.8	0.3	0.5
CaO	%	34.7	42.5	34.6	41.9	38.8
MgO	%	10.3	6.4	7.3	7.3	8.7
K_2_O	%	1.3	0.3	1	0.4	0.7
Na_2_O	%	0.4	0.3	0.2	0.2	0.4
Na_2_O eq	%	1.2	0.6	0.9	0.5	0.8
SO_3_	%	0.3	1.2	0.4	1.4	0.6
Chloride	%	nd	0.14	nd	nd	nd
Sulphide (S^2−^)	%	0.12	0.48	0.16	0.56	0.24
Moisture	%	0	8.7	2.6	9.1	0.2
*d*_50_	μm	53	41	26	36	31
Fineness	cm^2^/g	1768	2076	2668	2178	2407
Density	g/cm^3^	2.94	2.86	2.93	2.85	2.94

nd—non detectable.

**Table 2:** Correlation matrix (Pearson’s correlation) of test properties, reactivity indices, and chemical properties of GGBS.

		Compressive Strength, MPa			
		6 h	24 h	7 d	28 d
Basicity according to Tetmajer (CaO/SiO_2_)	Pearson’s Corr.	0.32	−0.57	0.19	0.93
	*p*-value	0.30	0.16	0.38	0.01
German Standard for “Eisenportlandzement” (CEM II-S)	Pearson’s Corr.	0.31	−0.34	0.30	0.85
	*p*-value	0.30	0.28	0.62	0.03
German Standard for “Hochofenzement” I	Pearson’s Corr.	0.20	−0.41	0.72	0.86
	*p*-value	0.37	0.25	0.27	0.03
German Standard for “Hochofenzement” II	Pearson’s Corr.	−0.18	0.74	−0.24	−0.84
	*p*-value	0.39	0.08	0.35	0.04
German Standard for special cements	Pearson’s Corr.	−0.07	−0.54	0.53	0.83
	*p*-value	0.46	0.18	0.18	0.04
F-value according to Keil	Pearson’s Corr.	0.04	−0.63	0.40	0.88
	*p*-value	0.48	0.13	0.25	0.03
Reactivity according to De Langavant	Pearson’s Corr.	0.05	−0.58	0.43	0.87
	*p*-value	0.47	0.15	0.24	0.03
Basicity according to Schwiete	Pearson’s Corr.	0.17	−0.66	0.29	0.91
	*p*-value	0.39	0.11	0.32	0.02
Reactivity according to Wang	Pearson’s Corr.	−0.01	−0.47	0.51	0.84
	*p*-value	0.49	0.21	0.19	0.04
European Standards for cement and blast-furnace slag I	Pearson’s Corr.	0.11	−0.46	0.43	0.86
	*p*-value	0.43	0.22	0.23	0.03
European Standards for cement and blast-furnace slag II	Pearson’s Corr.	0.52	0.01	0.20	0.60
	*p*-value	0.19	0.50	0.37	0.14
F-value according to Ehrenberg	Pearson’s Corr.	0.13	−0.54	0.37	0.90
	*p*-value	0.41	0.18	0.27	0.02
6 h Heat, J/g SCM (R^3^ test)	Pearson’s Corr.	0.75	−0.24	−0.39	0.84
	*p*-value	0.07	0.35	0.26	0.04
24 h Heat, J/g SCM (R^3^ test)	Pearson’s Corr.	0.84	−0.10	−0.46	0.73
	*p*-value	0.04	0.43	0.22	0.08
3 d Heat, J/g SCM (R^3^ test)	Pearson’s Corr.	0.84	0.07	−0.47	0.62
	*p*-value	0.04	0.46	0.21	0.13
7 d Heat, J/g SCM (R^3^ test)	Pearson’s Corr.	0.81	0.18	−0.46	0.55
	*p*-value	0.05	0.39	0.22	0.17
Bound water content, % (R^3^ test)	Pearson’s Corr.	0.97	0.05	−0.77	0.41
	*p*-value	0.00	0.47	0.07	0.25
CaO/Al_2_O_3_	Pearson’s Corr.	0.81	−0.15	−0.22	0.68
	*p*-value	0.05	0.40	0.36	0.10
CaO	Pearson’s Corr.	0.48	−0.53	0.06	0.90
	*p*-value	0.21	0.18	0.46	0.02
Al_2_O_3_	Pearson’s Corr.	−0.92	−0.37	0.53	−0.16
	*p*-value	0.01	0.27	0.18	0.40
SiO_2_	Pearson’s Corr.	0.05	0.57	−0.48	−0.86
	*p*-value	0.47	0.16	0.20	0.03
Moderate correlation					
High correlation					
Strong correlation					

## Data Availability

The original contributions presented in the study are included in the article, further inquiries can be directed to the corresponding author.

## Appendix A

**Table A1:** Reactivity indices according to the literature [9].

Reactivity Index	Definition	Criteria	S1	S2	S3	S4	S5
Basicity according to Tetmajer	$\frac{CaO}{SiO_{2}}$	-	0.91	1.20	0.87	1.14	1.09
German Standard for “Eisenportlandzement”	$\frac{CaO+MgO}{SiO_{2}+{Al}_{2}O_{3}}$	≥1	0.90	1.04	0.81	1.04	0.97
German Standard for “Hochofenzement” I	$\frac{CaO+MgO+\frac{1}{3}{Al}_{2}O_{3}}{SiO_{2}+{\frac{1}{3}Al}_{2}O_{3}}$	≥1	1.06	1.22	0.96	1.20	1.16
German Standard for “Hochofenzement” II	MnO	≤5 wt%	1.80	0.20	1.60	0.40	0.30
German Standard for special cements	$\frac{CaO+MgO+{Al}_{2}O_{3}}{SiO_{2}}$	≥1	1.50	1.71	1.35	1.64	1.71
F-value according to Keil	$\frac{CaO+CaS+\frac{1}{2}MgO+{Al}_{2}O_{3}}{SiO_{2}+MnO}$	>1.5	1.31	1.63	1.22	1.54	1.58
Reactivity according to De Langavant	${20+CaO+Al}_{2}O_{3}+\frac{1}{2}MgO-SiO_{2}$	-	33.85	42.00	30.45	39.75	41.05
Basicity according to Schwiete	$\frac{CaO+{Al}_{2}O_{3}-10}{SiO_{2}+10}$	-	0.77	0.97	0.73	0.92	0.93
Reactivity according to Wang	$\frac{CaO+MgO+{Al}_{2}O_{3}}{SiO_{2}+TiO_{2}}$	-	1.48	1.68	1.31	1.62	1.66
European Standards for cement and blast-furnace slag I	$\frac{CaO+MgO}{SiO_{2}}$	>1	1.18	1.38	1.05	1.34	1.33
European Standards for cement and blast-furnace slag II	$CaO+MgO+SiO_{2}$	>67 wt%	83.10	84.30	81.70	85.80	83.10
F-value according to Ehrenberg	$\frac{CaO+\frac{1}{2}MgO+\frac{1}{2}S^{-2}+{Al}_{2}O_{3}}{{SiO}_{2}+MnO+{{TiO}_{2}}^{2}}$	-	1.29	1.60	1.16	1.52	1.52
